# CLRM-13. INTRAOPERATIVE MICRODIALYSIS: GLIOMA INTELLIGENCE FROM BEHIND ENEMY LINES

**DOI:** 10.1093/noajnl/vdab112.012

**Published:** 2021-09-21

**Authors:** Cecile Riviere-Cazaux, Terry Burns

**Affiliations:** Mayo Clinic, MN, USA

## Abstract

**INTRODUCTION:**

Gliomas present a formidable challenge for translational progress. Heterogeneity within and between tumors may demand empirically individualized and adaptive paradigms requiring rapid mechanistic feedback. We asked if tumor-associated metabolic biomarkers from glioma extracellular fluid could impart mechanistic “intelligence” reflecting intra- and inter-tumoral heterogeneity.

**METHODS:**

Five live human gliomas (2 oligos; 2 IDH-WT GBMs; 1 IDH-mutant GBM), were evaluated in situ with high molecular weight (100kDA) intraoperative microdialysis using 3 disparately placed catheters. Isotonic 3% dextran perfusate was collected in 20 min (40mL) aliquots. CSF samples (n=21) were additionally evaluated from these and other patients with diverse brain tumors. The IDH-mutant glioma-associated oncometabolite D2-hydroxyglutarate (D2-HG) was quantified with targeted Liquid Chromotography-Mass Spectrometry (LC-MS). Over 200 metabolites were further evaluated via untargeted LC-MS using the Metabolon platform. Correlation, clustering, ROC and enrichment analyses were employed to identify correlations within and between patient samples.

**RESULTS:**

CSF samples from patients with IDH-mutant gliomas contained over twenty-fold higher levels of D2-HG (median 4.1 mM, range 1.6-13.2, n=7) compared to those from IDH-wild type tumors (median 0.19 mM; range 0.89-0.35, n=14). Microdialysate from IDH-mutant gliomas contained 10-953mM D2-HG, 9-63x higher than paired CSF samples. Interestingly, IDH status failed to predict the global metabolic signature of microdialysate. Microdialysate samples clustered into 2 major metabolic phenotype clusters with IDH-WT and IDH-mutant gliomas in each cluster. A superimposed metabolic signature distinguishing enhancing from non-enhancing tumor, was conserved in both patient clusters. Amino acid and carnitine metabolism predominated in microdialysate signatures. TCA cycle and Warburg-associated metabolites were differentially enriched in CSF samples after prior therapy independent of tumor burden.

**CONCLUSIONS:**

Intra-operative micro-dialysis may complement currently available “intelligence” regarding the phenotype, burden, and metabolism of live human gliomas and is feasible within standard-of-care surgical procedures. Future work will evaluate utility for pharmacodynamic feedback following novel early phase candidate therapies.

